# Exploring Factors Influencing Tooth Restorability Decisions Among Dental Students, Interns, and General Practitioners

**DOI:** 10.3290/j.ohpd.c_2338

**Published:** 2025-11-26

**Authors:** Alaa Redwan, Nouf Alsubhi, Ruzan Haider, Ola Kofiah, Salma Ghandourah, Danah Hammad, Rayan Sharka

**Affiliations:** a Alaa Redwan Assistant Professor, Preventive Dentistry Department, Faculty of Dental Medicine, Umm Al-Qura University, Makkah 24382, Saudi Arabia. Conceptualisation, formal analysis, data curation, writing – review and editing, supervision, project administration.; b Nouf Alsubhi General Dentist, Dental Teaching Hospital, Faculty of Dental Medicine, Umm Al-Qura University, Makkah 24382, Saudi Arabia. Methodology, investigation, data curation, writing – original draft preparation.; c Ruzan Haider General Dentist, Dental Teaching Hospital, Faculty of Dental Medicine, Umm Al-Qura University, Makkah 24382, Saudi Arabia. Methodology, investigation, data curation, writing – original draft preparation.; d Ola Kofiah General Dentist, Dental Teaching Hospital, Faculty of Dental Medicine, Umm Al-Qura University, Makkah 24382, Saudi Arabia. Methodology, investigation, data curation, writing – original draft preparation.; e Salma Ghandourah General Dentist, Dental Teaching Hospital, Faculty of Dental Medicine, Umm Al-Qura University, Makkah 24382, Saudi Arabia. Methodology, investigation, data curation, writing – original draft preparation.; f Danah Hammad General Dentist, Dental Teaching Hospital, Faculty of Dental Medicine, Umm Al-Qura University, Makkah 24382, Saudi Arabia. Methodology, investigation, data curation, writing – original draft preparation.; g Rayan Sharka Assistant Professor, Oral and Maxillofacial Surgery Department, Faculty of Dental Medicine, Umm Al-Qura University, Makkah 24382, Saudi Arabia. Conceptualisation, formal analysis, data curation, writing – review and editing, supervision.

**Keywords:** decision-making, dental education, dental extraction, preventive dentistry, teeth restorability

## Abstract

**Purpose:**

The decision to restore or extract a tooth is influenced by various clinical factors, but it remains subjective and lacks standardized guidance. This study evaluated the clinical decision-making capabilities of predoctoral students, interns, and general dental practitioners (GPs) regarding tooth restorability, exploring factors that affected their confidence in assessing tooth restorability.

**Materials and Methods:**

A cross-sectional study was conducted across multiple dental schools targeting predoctoral students, interns, and GPs. The online questionnaire included four hypothetical case scenarios for assessing decision-making regarding tooth restorability, as well as items assessing factors that influence confidence levels. The data were analyzed using chi-square tests, exploratory factor analysis (EFA), and regression analysis.

**Results:**

In total, 360 participants completed the questionnaire, with a response rate of 90%. There were statistically significant differences in some restorability and prognosis decisions across educational levels. EFA has identified two factors: preparedness for tooth restorability assessment and challenges and considerations in tooth restorability assessment. Regression analysis revealed that preparedness was a significant positive predictor of confidence (B = 0.791, p < 0.001), whereas challenges and considerations in tooth restorability assessment were not (B = –0.086, p = 0.165).

**Conclusion:**

Enhancing educational preparation can better equip dental practitioners to make confident and informed restorative decisions. Future research should explore strategies to enhance educational programs, mentorship, and guidelines to support dental practitioners in their decision-making processes.

The decision to extract or preserve a tooth is controversial and complex, requiring consideration not only from a restorative perspective by a multidisciplinary team, including periodontists, endodontists, and prosthodontists, but also of the patient’s expectations, preferences, and socioeconomic factors.^9,14,23,26^ Consequently, the dentist should perform a comprehensive examination and evaluation of the tooth and the remaining tooth structure to determine its restorability before initiating treatment.^19^ This evaluation includes several clinical and biological factors, such as the structural integrity of the remaining tooth (eg, amount of sound dentin and enamel, presence of cracks or fractures), the periodontal status (eg, attachment loss, pocket depth, bone support), and the endodontic prognosis (eg, presence of periapical pathology, canal morphology, previous root canal treatment outcomes).^9, 10,12^ Additionally, the occlusal load and the tooth’s role in the overall occlusion must be assessed, particularly in patients with parafunctional habits or malocclusion. Patient-centred considerations, including aesthetic demands, oral hygiene practices, compliance, and financial constraints, also play a critical role in the decision-making process.^12^

In dental practice, one of the biggest dilemmas is planning to extract a tooth with a questionable prognosis.^19^ Moreover, the decision to save or extract the first permanent molars (FPMs), which are the most prone to caries, is controversial in paediatric dentistry.^7^ FPMs are commonly extracted due to the high frequency of dental caries, extensive multiple surface restorations, pulpal symptoms, and developmental anomalies such as molar-incisor hypomineralisation.^1,24^

Previous studies have attempted to produce simplified and comprehensive guidelines to assist dental practitioners in making the best decisions regarding tooth preservation and extraction.^6,9,19,22^ Among these are structured restorability indices designed to standardise clinical judgement. The McDonald and Setchell Index evaluates restorability based on factors such as the amount of remaining dentine and the strategic importance of the tooth.^22^ The Dawood and Patel Dental Practicality Index (DPI) offers a more comprehensive and contemporary approach, incorporating clinical criteria, primarily the integrity of the tooth structure, periodontal condition, and endodontic assessment, to guide decision-making.^9^ Although some guidelines have demonstrated considerable efficacy, they have not been fully integrated into dental education due to subjective professional opinions, diverse clinical environments, varied educational backgrounds, and the time constraints inherent in clinical practice.^22^ Additionally, the rapid advancements in dental technology and techniques further complicate the standardisation of these guidelines across all clinical scenarios.^13^

Dental students and newly graduated dentists often lack formal education and training in assessing tooth restorability, thereby affecting their treatment decisions and confidence when interacting with patients.^3,10^ Also, several published studies have assessed dental students’ perceived confidence in performing various clinical procedures, including oral surgery, fixed and removable prosthodontics, endodontics, and operative dentistry.^2,5,8,28,30^ Al-Koky et al reported that final year dental students generally reported higher confidence in crown and bridge procedures compared to those in earlier years.^2^ The study also highlighted that clinical exposure and academic level were linked to confidence, whereas gender had little influence.^2^ In another study, Davey et al observed that students in advanced years felt more competent overall, yet many lacked confidence in performing root canal treatments, especially on molars with multiple roots,^8^ largely attributed to limited hands-on experience.^8^ Similarly, Shah et al reported that most students believed they had sufficient training in oral surgery, having completed a substantial number of extractions during their undergraduate years.^28^ However, there is a paucity of research investigating the confidence of dental students and newly graduated dentists in assessing teeth restorability, as well as the factors that may influence this confidence, including clinical experience, exposure to complex cases, availability and use of diagnostic tools, quality of undergraduate training, supervision and feedback, and access to continuing education opportunities.^4,5,15,17,28,29^ A proper understanding of these factors could enhance the development of targeted educational programmes and support systems designed to improve the confidence and decision-making abilities of future dental professionals.

Therefore, this study aimed to evaluate the ability of predoctoral students, interns, and GPs to make restorability decisions, exploring factors affecting their confidence in assessing tooth restorability. The null hypothesis was that there is no significant difference in the ability to assess tooth restorability among predoctoral students, interns, and GPs, and no significant association exists between their assessment levels and factors such as gender or education level.

## MATERIALS AND METHODS

### Ethical Considerations

The study was approved by the Biomedical Research Ethics Committee of Umm Al-Qura University (reference No. HAPO-02-K-012-2024-12-2374).

### Study Design and Population

This cross-sectional study was conducted at multiple dental schools across Saudi Arabia from December 2024 to March 2025. The participants included dental students in their clinical years (fifth- and sixth-year undergraduates), interns in their clinical training year programme, and dentists who had graduated within the past five years and were recruited via convenience sampling. The exclusion criteria were dental students in their preclinical years and dentists who had graduated more than five years ago. The sample size was calculated with a 5% margin of error, a 95% confidence level, a population size of 5,000, and a response distribution of 50% using the Raosoft online sample size calculator; the minimum recommended sample size was 357 participants.

### Study Procedures

An online questionnaire, consisting of four sections, was developed and piloted with 15 dental students to detect issues and ensure that scenarios and questions were unambiguous. No major issues in wording and structure were identified.

The first section provided information about the research study, including its objectives and procedures, to ensure that participants were fully informed before giving their consent to participate. The second section was designed to collect general demographic information, including gender, age, level of education, and affiliations with dental schools or dental practices. The third section presented four hypothetical clinical scenarios: two cases involving adult patients and two cases involving paediatric patients. The participants were then asked to make decisions regarding the restorability and prognosis of the tooth in each scenario. Considering the subjectivity of determining the restorability decision, a team of five consultants specialising in prosthodontics, endodontics, restorative, periodontics, and paediatrics was invited to review the four cases for clarity, providing feedback to ensure that the clinical scenarios were clear and comprehensible. The adult scenarios included clinical images, intraoral photographs, periapical/bitewing radiographs, and a patient history, whereas the paediatric cases included an orthopantomogram (OPG) and periapical radiographs.

**Fig 1:**
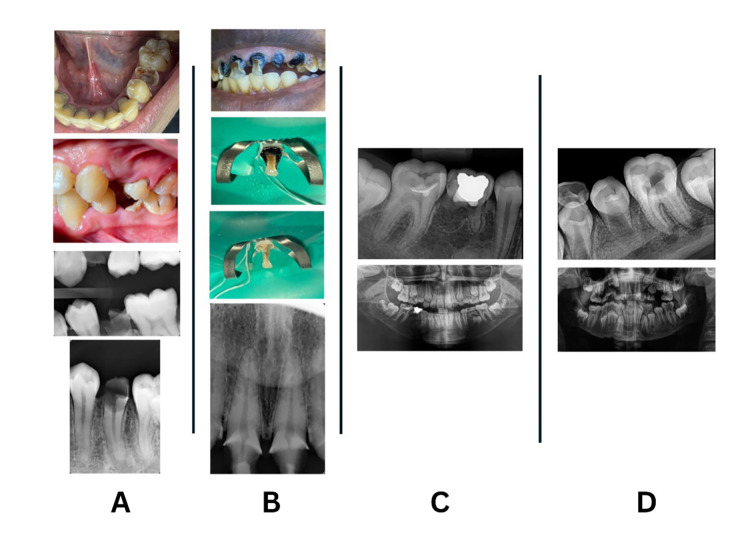
Photos of clinical scenarios.

The first clinical scenario involved a healthy 22-year-old patient who presented with multiple severely damaged teeth, necessitating a comprehensive treatment plan. The endodontic diagnosis for the mandibular left premolar (#35) was necrotic pulp with symptomatic apical periodontitis. Following caries excavation, the biological width was determined to be 1 mm circumferentially. This case was scored as Level 6 according to the Dawood and Patel Restorability Index (Fig 1a).

The second clinical scenario involved a 35-year-old patient with severely damaged anterior teeth. The endodontic diagnosis for the maxillary left central incisor (#21) was reversible pulpitis with normal apical tissue. The biological width was determined to be 3 mm circumferentially. This case was scored as Level 2 according to the Dawood and Patel Restorability Index (Fig 1b).

The third clinical scenario involved an 11-year-old patient with remnants of a previously placed restoration in the mandibular right first molar (#46). The endodontic diagnosis was reversible pulpitis with normal apical tissue. This case was scored as Level 1 according to the Dawood and Patel Restorability Index (Fig 1c).

The fourth clinical scenario involved a 9-year-old patient whose mandibular left first molar (#36) had caries that approximated the pulp, with no remaining tooth structure on the buccal wall. The endodontic diagnosis for tooth #36 was necrotic pulp with acute apical periodontitis. This case was scored as Level 6 according to the Dawood and Patel Restorability Index (Fig 1d).

The final section included eight questions adopted from a previous study to identify factors that influence the participants’ confidence levels in assessing tooth restorability.^3^ These questions were measured on a five-point Likert scale, ranging from ‘strongly disagree’ to ‘strongly agree’.

### Statistical Analysis

Statistical analysis was performed using SPSS software version 29 for Windows (IBM, New York, NY, USA). Categorical variables were summarised using frequencies and percentages (%), with the Chi-square test applied to determine the association between groups. The suitability of the data set for exploratory factor analysis (EFA) was evaluated using the Kaiser–Meyer–Olkin (KMO) measure of sampling adequacy and Bartlett’s test of sphericity. For the data to meet the assumptions required for EFA, KMO values must exceed 0.50, and the significance level of Bartlett’s test should be less than 0.05.^16^ Communalities were calculated to determine the proportion of variance in each variable that could be explained by the extracted factors. The number of factors was determined based on eigenvalues, the scree plot, and established theoretical frameworks.^16^ Orthogonal rotation, specifically varimax rotation, was applied to achieve a simpler and more interpretable factor structure.^16^ Cronbach’s alpha was calculated to assess the internal consistency and reliability of the factors. Additionally, a multiple regression analysis was conducted to examine the impact of the identified factors on confidence levels. Multicollinearity was assessed using the variance inflation factor (VIF) analysis, with values below 10 indicating the absence of multicollinearity. Also, the standard error of the estimate was used to measure the accuracy of predictions, and the Durbin–Watson statistic was employed to check for autocorrelation in the residuals. Results were evaluated at a significance level of P <0.05.

## RESULTS

### Subject Characteristics

In total, the questionnaire was sent to 400 participants, of which 360 completed the survey, resulting in a response rate of 90%. Of these, 187 (51.9%) were female, while 173 (48.1%) were male, with a median age of 24 years. Participants represented various educational levels, with 149 (41.4%) being predoctoral students, 84 (23.3%) interns, and 127 (35.3%) GPs. Furthermore, 150 (41.7%) of the respondents pursued their dental education at Umm Al-Qura University, while the remaining respondents were from different universities.

#### Association between restorability decision and prognosis of the clinical scenarios with gender and level of education

Table 1 highlights the associations between gender and restorability decisions across various clinical scenarios. There was a statistically significant association in scenario 1, where the restorability decision significantly correlated with gender (P = 0.010). Specifically, a higher proportion of males (91.9%) deemed the scenario restorable compared to females (82.9%).

**Table 1:** The association between restorability decision and prognosis with gender

	Gender
Male	Female	Total	P value
Decision of scenario 1 (#21)	Restorable	159 (91.9)	155 (82.9)	314 (87.2)	0.010*
Non-restorable	14 (8.1)	32 (17.1)	46 (12.8)
Prognosis of scenario 1 (#21)	Favourable	65 (37.6)	50 (26.7)	115 (31.9)	0.065
Questionable	98 (56.6)	120 (64.2)	218 (60.6)
Hopeless	10 (5.8)	17 (9.1)	27 (7.5)
Decision of scenario 2 (#35)	Restorable	25 (14.5)	34 (18.2)	59 (16.4)	0.339
Non-restorable	148 (85.5)	153 (81.8)	301 (83.6)
Prognosis of scenario 2 (#35)	Favourable	25 (14.5)	17 (9.1)	42 (11.7)	0.030*
Questionable	26 (15)	47 (25.1)	73 (20.3)
Hopeless	122 (70.5)	123 (65.8)	245 (68.1)
Decision of scenario 3 (#46)	Restorable	122 (70.5)	114 (61)	236 (65.6)	0.057
Non-restorable	51 (29.5)	73 (39)	124 (34.4)
Prognosis of scenario 3 (#46)	Favourable	112 (64.7)	108 (57.8)	220 (61.1)	0.229
Questionable	27 (15.6)	28 (15)	55 (15.3)
Hopeless	34 (19.7)	51 (27.3)	85 (23.6)
Decision of scenario 4 (#36)	Restorable	147 (85)	154 (82.4)	301 (83.6)	0.503
Non-restorable	26 (15)	33 (17.6)	59 (16.4)
Prognosis of scenario 4 (#36)	Favourable	111 (64.2)	114 (61)	225 (62.5)	0.774
Questionable	45 (26)	51 (27.3)	96 (26.7)
Hopeless	17 (9.8)	22 (11.8)	39 (10.8)
*P < 0.05

Regarding prognosis, a significant association was observed in scenario 2 (P = 0.030), with more males (14.5%) considering the prognosis favourable compared to females (9.1%).

Table 2 shows the significant associations between education level and restorability decisions in scenarios 2 and 4. In scenario 2, the restorability decision significantly correlated with education level (P = 0.018), with a higher proportion of predoctoral students (22.1%) considering scenario 2 restorable compared to interns (16.7%) and GPs (9.4%). Similarly, there was a significant association between the level of education and restorability decision in scenario 4 (P = 0.016), with more GPs (72.4%) deeming the scenario restorable compared to interns (70.2%) and predoctoral students (57%). Regarding the prognosis, there was a significant association observed between the level of education and prognosis in scenarios 1 (P = 0.019) and 4 (P = 0.012), with more GPs considering the prognosis favourable compared to interns and predoctoral students.

**Table 2:** The association between restorability decision and prognosis with level of education

	Level of education
Predoctoral students	Interns	GPs	Total	P value
Decision of scenario 1 (#21)	Restorable	123 (82.6)	76 (90.5)	115 (90.6)	314 (87.2)	0.083
Non-restorable	26 (17.4)	8 (9.5)	12 (9.4)	46 (12.8)
Prognosis of scenario 1 (#21)	Favourable	35 (23.5)	28 (33.3)	52 (40.9)	115 (31.9)	0.019*
Questionable	98 (65.8)	51 (60.7)	69 (54.3)	218 (60.6)
Hopeless	16 (10.7)	5 (6)	6 (4.7)	27 (7.5)
Decision of scenario 2 (#35)	Restorable	33 (22.1)	14 (16.7)	12 (9.4)	59 (16.4)	0.018*
Non-restorable	116 (77.9)	70 (83.3)	115 (90.6)	301 (83.6)
Prognosis of scenario 2 (#35)	Favourable	23 (15.4)	10 (11.9)	9 (7.1)	42 (11.7)	0.088
Questionable	35 (23.5)	17 (20.2)	21 (16.5)	73 (20.3)
Hopeless	91 (61.1)	57 (67.9)	97 (28.8)	245 (68.1)
Decision of scenario 3 (#46)	Restorable	85 (57)	59 (70.2)	92 (72.4)	236 (65.6)	0.016*
Non-restorable	64 (43)	25 (29.8)	35 (27.6)	124 (34.4)
Prognosis of scenario 3 (#46)	Favourable	75 (50.3)	56 (66.7)	89 (70.1)	220 (61.1)	0.012*
Questionable	30 (20.1)	10 (11.9)	15 (11.8)	55 (15.3)
Hopeless	44 (29.5)	18 (21.4)	23 (18.1)	85 (23.6)
Decision of scenario 4 (#36)	Restorable	122 (81.9)	67 (79.8)	112 (88.2)	301 (83.6)	0.204
Non-restorable	27 (18.1)	17 (20.2)	15 (11.8)	59 (16.4)
Prognosis of scenario 4 (#36)	Favourable	90 (60.4)	52 (61.9)	83 (65.4)	225 (62.5)	0.848
Questionable	40 (26.8)	23 (27.4)	33 (26)	96 (26.7)
Hopeless	19 (12.8)	9 (10.7)	11 (8.7)	39 10.8)
*P < 0.05

#### Exploring factors influencing predoctoral students, interns, and GPs in making confident restorability decisions

**Fig 2:**
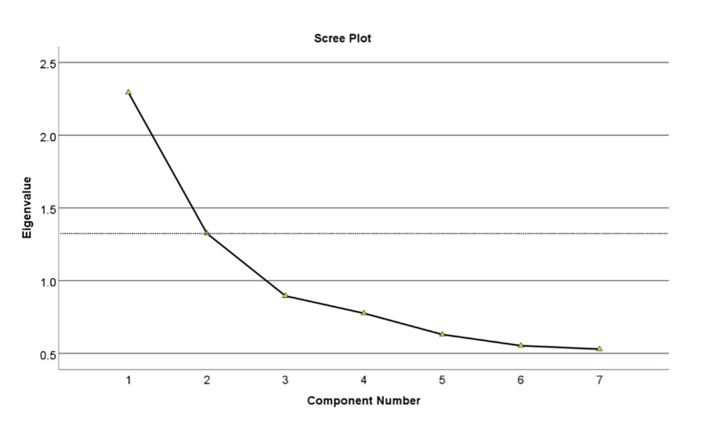
A scree plot analysis of exploratory factor analysis.

The data were suitable for EFA factor extraction, as indicated by a KMO of 0.700 and a significant Bartlett’s test of sphericity (χ^2^(21) = 336.703, P <0.001). All item communalities exceeded 0.40, suggesting that each item shared a substantial proportion of variance with the others. Two factors were extracted based on eigenvalues greater than 1 and the scree plot (Fig 2). These factors included a total of seven items and accounted for 51.68% of the total variance. The reliability of these factors was assessed using Cronbach’s alpha (α), with factor 1 yielding an α of 0.629 and factor 2 yielding an α of 0.600 (Table 3). Based on the grouping of items and their respective loadings, factor 1 was labelled as ‘preparedness for tooth restorability assessment’, reflecting the respondents’ perceived adequacy of their educational and clinical preparation. Factor 2 was labelled as ‘Challenges and considerations in tooth restorability assessment’, encapsulating the various obstacles and considerations that influence their decision-making (Table 4).

**Table 3:** The seven items identified from exploratory factor analysis using principal component extraction are displayed in the rotated component matrix from highest to lowest variance

Items	loading	h^2^	Mean (St. Deviation)	Cronbach’s alpha (α)
Factor I	Factor II
Educational preparation	0.785		0.616	3.97 (0.876)	0.629
Clinical experience	0.772		0.618	4.00 (0.831)
Mentorship	0.661		0.445	4.17 (0.866)
Inconsistent guidelines		0.801	0.642	3.81 (0.921)	0.600
Independent work challenges		0.639	0.411	4.08 (0.843)
Patient preferences		0.606	0.437	3.98 (0.954)
Time constraints		0.587	0.448	3.86 (0.864)
Explained variance (%)	26.101	25.586			
Extraction method: Principal component analysis.Rotation method: Varimax with Kaiser normalisation.α Rotation converged in three iterations.

**Table 4:** The extracted factors with descriptions and labels

Factors	Description	Labelling
Factor I	This factor reflects participants’ perceived readiness to assess tooth restorability, encompassing their educational background, mentorship experiences, and clinical practice. It includes the effectiveness of undergraduate courses in preparing them for this task, the pivotal role of mentorship and guidance from experienced dentists in developing their skills, and the adequacy of their clinical experience in building confidence and competence in assessing tooth restorability.	Preparedness for tooth restorability assessment
Factor II	This factor captures the various obstacles and contextual considerations encountered by participants during the assessment of tooth restorability. It includes challenges related to independent clinical decision-making, the integration of patient preferences, the influence of time constraints in practice settings, and the complications arising from inconsistent guidelines. Collectively, these elements underscore the complexity and multifactorial nature of the decision-making process involved in evaluating tooth restorability.	Challenges and considerations in tooth restorability assessment


#### Assessing factors influencing predoctoral students, interns, and GPs in making confident restorability decisions

**Fig 3:**
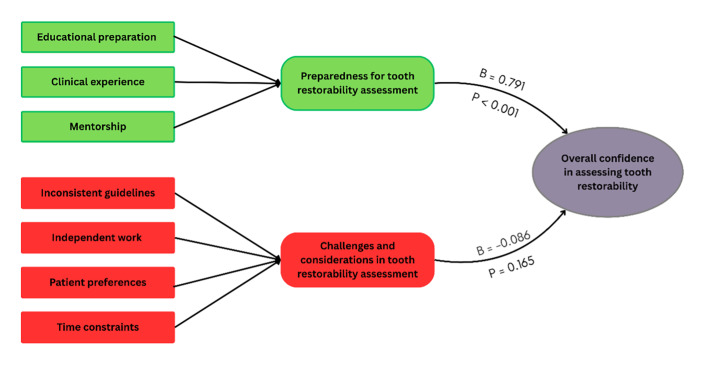
A summary chart of EFA and multiple regression analysis.

Multiple regression analysis conducted to examine the impact of these factors on the overall confidence in assessing tooth restorability was statistically significant [F(2, 357) = 99.080, P <0.001] and explained approximately 35.7% of the variance in overall confidence (R^2^ = 0.357). The adjusted R^2^ value of 0.353 indicates a good fit, and the Durbin–Watson statistic was 1.669, indicating no severe autocorrelation issues in the residuals (Table 5). ‘Preparedness for tooth restorability assessment’ was a significant positive predictor of overall confidence in assessing tooth restorability (B = 0.791, P <0.001) in contrast to ‘challenges and considerations in tooth restorability assessment’ (Table 6 and Fig 3).

**Table 5:** Regression analysis model summary

Model summary^b^
Model	R	R square	Adjusted R square	Std. error of the estimate	Change statistics	Durbin–Watson
R square change	F change	df1	df2	Sig. F change
1	0.597^a^	0.357	0.353	0.676	0.357	99.080	2	357	<0.001	1.669
a. Predictors: (Constant), Preparedness for tooth restorability assessment, Challenges and considerations in tooth restorability assessment.b. Dependent variable: Overall confidence in assessing tooth restorability.

**Table 6:** Regression analysis coefficients

Model	Coefficients^a^	t	Sig.
Unstandardised coefficients	Standardised coefficients
B	Std. Error	Beta
(Constant)	1.076	0.286		3.762	<0.001*
Challenges and considerations in tooth restorability assessment	–0.086	0.062	–0.062	–1.392	0.165
Preparedness for tooth restorability assessment	0.791	0.057	0.612	13.832	<0.001*
a. Dependent variable: overall confidence in assessing tooth restorability.*P < 0.05

## DISCUSSION

This study evaluated dental students’, interns’, and GPs’ clinical reasoning skills and decision-making process when faced with a complex question of whether to restore or extract the tooth. Factors affecting their confidence in assessing teeth restorability were also explored to determine whether their level of education and gender influence restorability and prognosis assessment.

The study revealed no significant differences in restorability and prognosis decisions based on gender across most clinical scenarios, suggesting that gender did not consistently influence decision-making among respondents, in line with previous studies.^3,25^ While some literature has suggested potential gender-based tendencies in treatment preferences, such as a greater inclination among male dentists to extract and place implants,^18^ the current study did not find consistent evidence to support such differences.

In contrast, the education level impacted restorability and prognosis decisions in our cohort. Lang-Hua et al explored how dental practitioners decide whether to extract or maintain questionable teeth,^21^ reporting that decisions varied according to the practitioners’ training, with general dental practitioners being more likely to extract compared to specialists or those with postgraduate training. This also supports the hypothesis that advanced clinical competence, developed through increased educational and practical experience, influences the decision-making process. In a related study, Tolentino et al also observed that more experienced dentists tended to favour tooth extraction.^31^ However, some studies have reported no significant association between education level and restorability decisions.^3,25^ This variation suggests that while education and training can influence decision-making, other factors such as clinical experience, personal preferences, and specific case circumstances could also be important.

There was more variability in the respondents’ decisions regarding the paediatric scenarios, emphasising the complexity of treating younger patients. Unlike adults, restorability decisions in children are influenced by developmental factors such as root maturity, the presence of successor teeth, and the potential for spontaneous space closure, all of which significantly impact whether a tooth should be restored or extracted.^20^ Sometimes, extracting a tooth might support better long-term outcomes, such as proper alignment or easier orthodontic planning. These findings suggest that patient age adds complexity to restorability assessments, as dentists must think beyond the condition of the tooth and consider growth, development, and the child’s future dental health.^24^

Two key factors that influence confidence in restorability decisions among participants were identified: preparedness for tooth restorability assessment and challenges and considerations in tooth restorability assessment. Preparedness for tooth restorability assessment encompasses items such as educational preparation, clinical experience, and mentorship, highlighting the crucial role of comprehensive training and support in building confidence among dental practitioners.^8,15^ Dental education provides foundational knowledge, while clinical experience enables practitioners to apply this knowledge in practical settings.^8,15,29,30^ Mentorship provides guidance and support, helping practitioners navigate complex cases and enhance their decision-making confidence.^11,30^

Challenges and considerations in tooth restorability assessment reflect the diverse nature of several factors that impact restorability decisions. These include inconsistent guidelines, the necessity for independent work, patient preferences, and time constraints. Inconsistent guidelines can create uncertainty and variability in decision-making, while independent work requires practitioners to rely on their judgement and expertise. Patient preferences must be taken into account to ensure patient-centred care, and time constraints during the clinical routine may pressure dental practices to compromise on the thoroughness of assessments. These findings support existing literature that highlights the complexity of restorability decisions, considering that focusing on enhancing preparedness and reducing challenges faced is essential for improving confidence in restorability decisions.^3,5,27^ A study conducted in the UK highlighted that students tend to feel less confident with more complex procedures, which are less frequently practised during their undergraduate education.^15^

The preparedness for tooth restorability assessment factor significantly positively impacted the participants’ confidence in restorability decisions. This underscores the essential role of comprehensive educational preparation, clinical experience, and effective mentorship in building confidence among dental practitioners. Robust training programmes that thoroughly prepare practitioners can result in more confident and consistent decision-making. This finding aligns with previous studies that highlight the importance of both theoretical and clinical education in enabling students to perform dental procedures with greater confidence.^4,5,15,17,28,29^ Generally, dental students felt well-prepared for basic clinical procedures and communication skills commonly practised during their undergraduate education, but may lack confidence in orthodontic assessments, treatment planning, and crowns. Therefore, these areas require further training and experience to improve students’ confidence levels.^4^

Conversely, the factor related to challenges and considerations in tooth restorability assessment showed a negative correlation with confidence levels, although this relationship was not statistically significant. This suggests that while obstacles such as inconsistent guidelines, the need for independent work, patient preferences, and time constraints may present challenges, they do not substantially erode dental students’ and practitioners’ confidence. The solid foundation established by preparedness may help to counteract the adverse effects of these challenges.^29,30^

Although this study was conducted across multiple dental institutions in Saudi Arabia, future research should include participants from other countries to gain broader insights and facilitate cross-cultural comparisons. Expanding the geographic scope of the study can help identify potential differences and similarities in restorability decision-making across diverse populations. Additionally, restorability decisions and prognosis assessments were based on hypothetical case scenarios in which the clinical and radiographic images may be unclear, which may not fully replicate the complexity of clinical practice. Confidence levels were also self-reported, introducing the potential for response bias. Other factors, such as prior clinical exposure, mentorship quality, and curricular differences, were not evaluated and may have influenced outcomes. Furthermore, although some participants may have been familiar with the Dawood and Patel Restorability Index, it was not formally explained before the scenario evaluations. This lack of standardisation in understanding the index may have influenced how participants assessed restorability and should be acknowledged as a limitation.

This study highlights that predoctoral students must have better clinical training, exposure to hypothetical restorability dilemmas, knowledge about the restorability decision factors and scoring indexes, and improve their critical thinking. Also, they should be aware of the multidisciplinary scope of restoration and the necessity of evaluating each case from a variety of different aspects and perspectives. Furthermore, research regarding restorability in paediatrics is ongoing, and there should be greater emphasis placed on the second primary molar replacing the extracted FPM, an option that was not considered.

## CONCLUSIONS

Strengthening educational preparation and structured mentorship can enhance dental practitioners’ confidence in restorability decision-making. Future research should explore strategies to optimise training and educational programmes that support sound clinical judgement.

### Acknowledgements

#### Ethical approval and consent to participate

The study received approval from the Biomedical Research Ethics Committee of Umm Al-Qura University, with reference no. HAPO-02-K-012-2024-12-2374.

All participants gave informed written consent before participating in this study.

#### Funding

This research did not receive any specific grant from funding agencies in the public, commercial, or not-for-profit sectors.

#### Institutional review board statement

The study received approval from the Biomedical Research Ethics Committee of Umm Al-Qura University, with reference No. HAPO-02-K-012-2024-12-2374.

## References

[ref11] Fallatah HI, Soo Park Y, Farsi J, Tekian A (2018). Mentoring clinical-year medical students: factors contributing to effective mentoring. J Med Educ Curric Dev.

